# The Impact of Vitamin E Intake on Cardiometabolic Outcomes of Testosterone Replacement Therapy in Men with Late-Onset Hypogonadism

**DOI:** 10.3390/ijms27208953

**Published:** 2026-10-09

**Authors:** Robert Krysiak, Witold Szkróbka, Karolina Kowalcze, Grzegorz Machnik, Karolina Zapletal-Pudełko, Bogusław Okopień

**Affiliations:** 1 Department of Internal Medicine and Clinical Pharmacology, Medical University of Silesia, Medyków 18, 40-752 Katowice, Poland; 2 Department of Pediatrics in Bytom, Faculty of Health Sciences in Katowice, Medical University of Silesia, Stefana Batorego 15, 41-902 Bytom, Poland; kkowalcze@sum.edu.pl; 3 Department of Pathophysiology, Faculty of Medicine, Academy of Silesia, Rolna 43, 40-555 Katowice, Poland; 4 Department of Molecular Biology, Medical University of Silesia, 40-752 Katowice, Poland

**Keywords:** cardiometabolic risk, late-onset hypogonadism, men’s health, testosterone therapy, vitamin E

## Abstract

Androgen deficiency is associated with an increased risk of cardiometabolic complications, whereas vitamin E may exert protective effects against cardiovascular and metabolic diseases. This pilot prospective cohort study examined whether cardiometabolic outcomes observed during exogenous testosterone therapy differed according to vitamin E intake in men with insufficient endogenous testosterone production. Three cohorts of men with late-onset hypogonadism (LOH) (n = 29 per group), matched for age, smoking status, body mass index, and baseline testosterone concentration, were classified according to vitamin E intake as low, adequate, or high; 29 men without LOH served as controls. Men with LOH received intramuscular testosterone injections every three weeks for six months. Cardiometabolic parameters were assessed before and after treatment, including biochemical markers, carotid intima–media thickness, retinal vascular sclerotic changes, and estimated 10-year cardiovascular risk. Compared with controls, men with LOH had higher HOMA-IR, LDL cholesterol, uric acid, fibrinogen, homocysteine, high-sensitivity C-reactive protein (hsCRP), urinary albumin-to-creatinine ratio (UACR), carotid intima–media thickness, and estimated 10-year cardiovascular risk. Elevations in homocysteine, hsCRP, and UACR were more pronounced in men with low or high vitamin E intake than in those with adequate intake. Testosterone levels increased during treatment in all groups. Improvements were observed in most cardiometabolic parameters in men with adequate vitamin E intake, whereas changes were limited to HOMA-IR in those with low intake. In men with high intake, no significant changes were observed except for a decrease in HDL cholesterol. At study completion, carotid intima–media thickness and retinal vascular sclerotic changes in men with adequate vitamin E intake did not differ significantly from those in controls. Changes in assessed risk factors correlated with increases in testosterone levels and mean daily vitamin E intake. These findings suggest an association between vitamin E intake and cardiometabolic outcomes observed during testosterone therapy in men with testosterone deficiency.

## 1. Introduction

In contrast to the ovaries, the decline in testicular hormonal function is gradual. It typically begins around age 40, with an estimated annual decrease of approximately 1.6% in total testosterone and 2–3% in free and bioavailable testosterone [1]. Because of this slow decline, clinical manifestations of testosterone deficiency later in life are generally less pronounced than those observed in postmenopausal women or in men with congenital or early-onset hypogonadism [2,3]. In some individuals—estimated at 30–40% of men aged 40–70—the decline may be more pronounced, leading to late-onset hypogonadism (LOH) [4,5]. Manifestations of LOH include impaired sexual function, reduced muscle mass and strength, osteopenia or osteoporosis, cognitive decline, increased visceral fat accumulation, and decreased insulin sensitivity [3]. Low testosterone levels have also been associated with an increased risk of endothelial dysfunction, low-grade systemic inflammation, hypercoagulability, and more pronounced atherosclerotic changes in the vascular wall [2,4]. Several studies have shown a correlation between low testosterone levels and increased risk of cardiovascular mortality, as well as a higher incidence of cardiovascular disease, diabetes, and metabolic syndrome [6,7,8]. These observations suggest potential benefits of testosterone replacement therapy. Despite this association, interventional studies do not permit definitive conclusions regarding the cardiometabolic effects of exogenous testosterone. Some studies reported reductions in all-cause mortality, stroke, and myocardial infarction [9,10]; however, other studies found no such effects [11], and in some cases, testosterone therapy was associated with an increased risk of adverse cardiovascular outcomes [12]. Similarly, improvements in carbohydrate metabolism were observed in only some studies [13], whereas others reported no significant effect [14]. Although these discrepancies may reflect differences in study populations, the influence of environmental factors cannot be excluded. We recently demonstrated such a role for vitamin D, showing that deficiency impairs the cardiometabolic effects of exogenous testosterone—an effect that normalizes upon restoration of adequate vitamin D status [15].

The term “vitamin E” refers to a group of eight plant-derived, fat-soluble compounds, including four tocopherols and four tocotrienols, with α-tocopherol exhibiting the greatest biological activity [16,17]. This vitamin possesses potent antioxidant properties and can scavenge free radicals, thereby reducing oxidative stress and limiting oxidative damage [16]. Preclinical investigations have demonstrated that it also slows low-density lipoprotein (LDL) oxidation, suppresses smooth muscle cell proliferation, and reduces platelet adhesion and aggregation [18,19]. Additionally, it has been shown to inhibit the expression and activity of adhesion molecules, decrease leukotriene synthesis, and enhance prostacyclin release. Collectively, these antioxidant and other pleiotropic effects may contribute to protective effects against the development of atherosclerosis [19,20]. Results from clinical trials evaluating exogenous vitamin E supplementation have yielded inconsistent findings. A reduction in the risk of cardiovascular mortality and nonfatal myocardial infarction has been observed among individuals with angiographically confirmed coronary atherosclerosis [21], as well as a decrease in the composite endpoint (including myocardial infarction, ischemic stroke, peripheral vascular disease, and unstable angina) and in myocardial infarction alone among hemodialysis patients with coexisting cardiovascular disease [22]. However, these findings have not been corroborated by other investigators whose studies included individuals both with and without diagnosed coronary artery disease [23,24]. Moreover, a meta-analysis of 38 randomized controlled trials involving 2171 patients with diabetes demonstrated that vitamin E supplementation was associated with significant reductions in glycated hemoglobin (HbA_1c_), fasting insulin levels, and the homeostatic model assessment of insulin resistance (HOMA-IR) among individuals with diabetes [25]. Conversely, a meta-analysis encompassing 19 clinical trials and 135,967 participants demonstrated that long-term administration of high-dose vitamin E (exceeding 400 IU per day) is associated with increased all-cause mortality [26].

To date, only a limited number of studies have examined the relationship between vitamin E and testicular function. In rats, vitamin E administration has been shown to protect spermatozoa and spermatogenic epithelial cells under conditions associated with impaired testicular function [27]. Moreover, vitamin E deficiency has been associated with elevated luteinizing hormone (LH) levels and reduced testosterone levels [28], whereas exogenous vitamin E administration has been shown to increase testosterone concentrations in both testicular tissue and plasma [29]. Vitamin E has also been shown to inhibit the formation of aortic fatty lesions in castrated rats in a dose-dependent manner [30]. In humans, serum α-tocopherol levels have been inversely correlated with testosterone and androstenedione levels [31,32]. Moreover, long-term α-tocopherol supplementation in elderly men has been shown to reduce serum androgen concentrations, including testosterone and androstenedione [33]. These conflicting findings underscore the uncertainty surrounding the relationship between vitamin E and male gonadal function, while the reasons for these discrepancies remain unclear and have not been adequately addressed in previous studies.

Approximately 92% of men in the United States are estimated to have dietary α-tocopherol intakes below the Estimated Average Requirement (EAR) [34]. Although vitamin E intake among European men is slightly higher, it remains relatively low (13 mg/day) [35]. Conversely, NHANES 1999–2000 data indicated that 11.3% of adults consumed vitamin E supplements at doses ≥ 400 IU/day, with higher prevalence among older individuals [36]. Although high-dose supplementation has likely declined, chronic exposure to such doses may remain relevant because they are still available in some dietary supplements. Testosterone replacement therapy represents a clinically important exposure among middle-aged and older men [37]. Therefore, many men may receive testosterone therapy in the setting of low vitamin E intake, whereas others may be exposed to both testosterone therapy and high-dose vitamin E supplementation. As the relationship between vitamin E intake and cardiometabolic outcomes during testosterone therapy remains unclear, this study examined whether these outcomes differed according to vitamin E intake in middle-aged and older men.

## 2. Results

**Figure 1:**
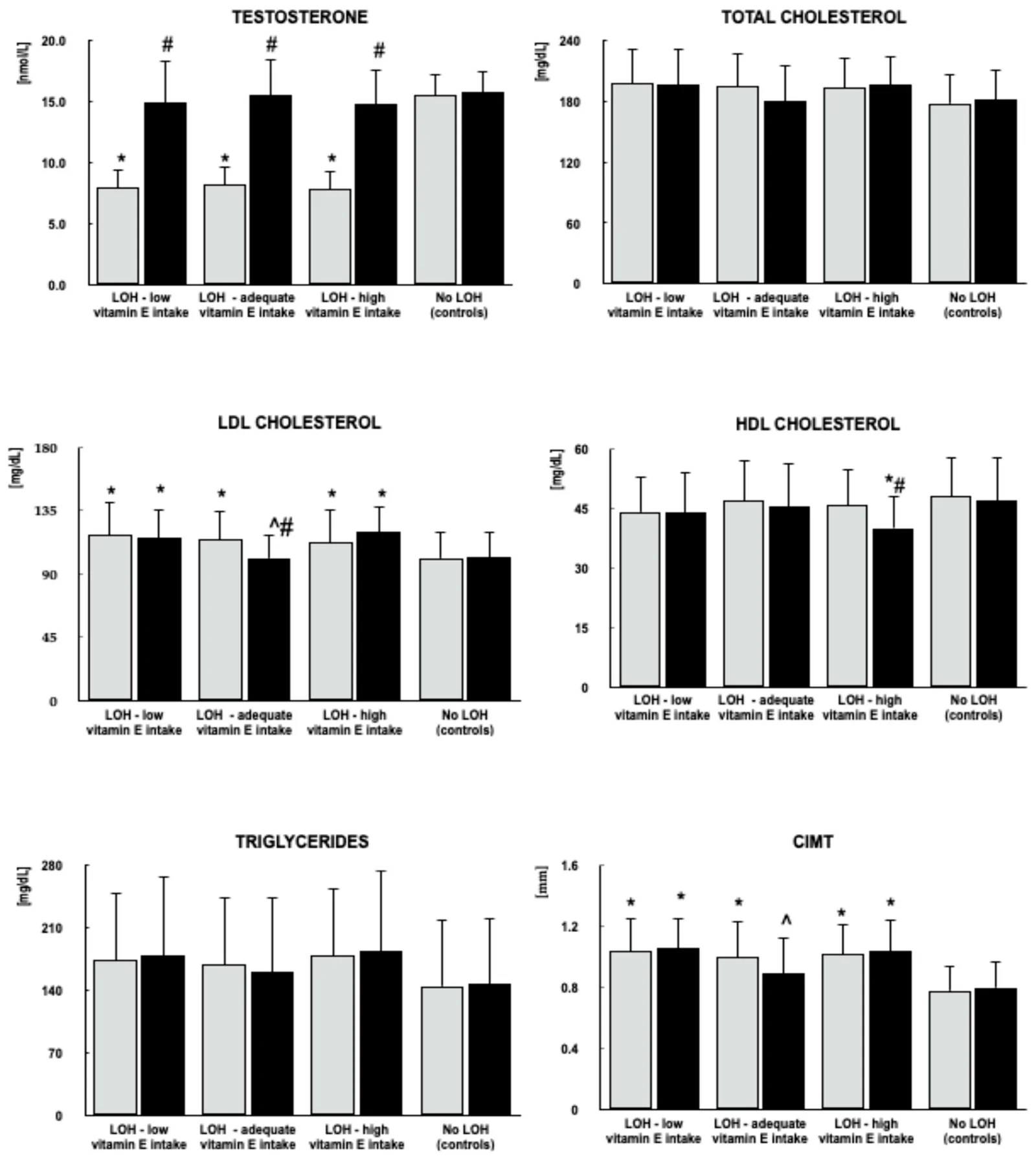
Effects of testosterone replacement on testosterone, lipids and carotid intima–media thickness in men with LOH, stratified by vitamin E intake. Data are presented as mean and standard deviation. Gray bars represent values at the beginning of the study, while black bars represent values at study completion. Post hoc comparisons: * *p* < 0.05 vs. value at the corresponding time point in men without LOH; ^^^ *p* < 0.05 vs. values at the corresponding time point in men with LOH with low or high vitamin E intake; ^#^ *p* < 0.05 vs. baseline value within the same group. *p*-values were adjusted for multiple comparisons.

**Figure 2:**
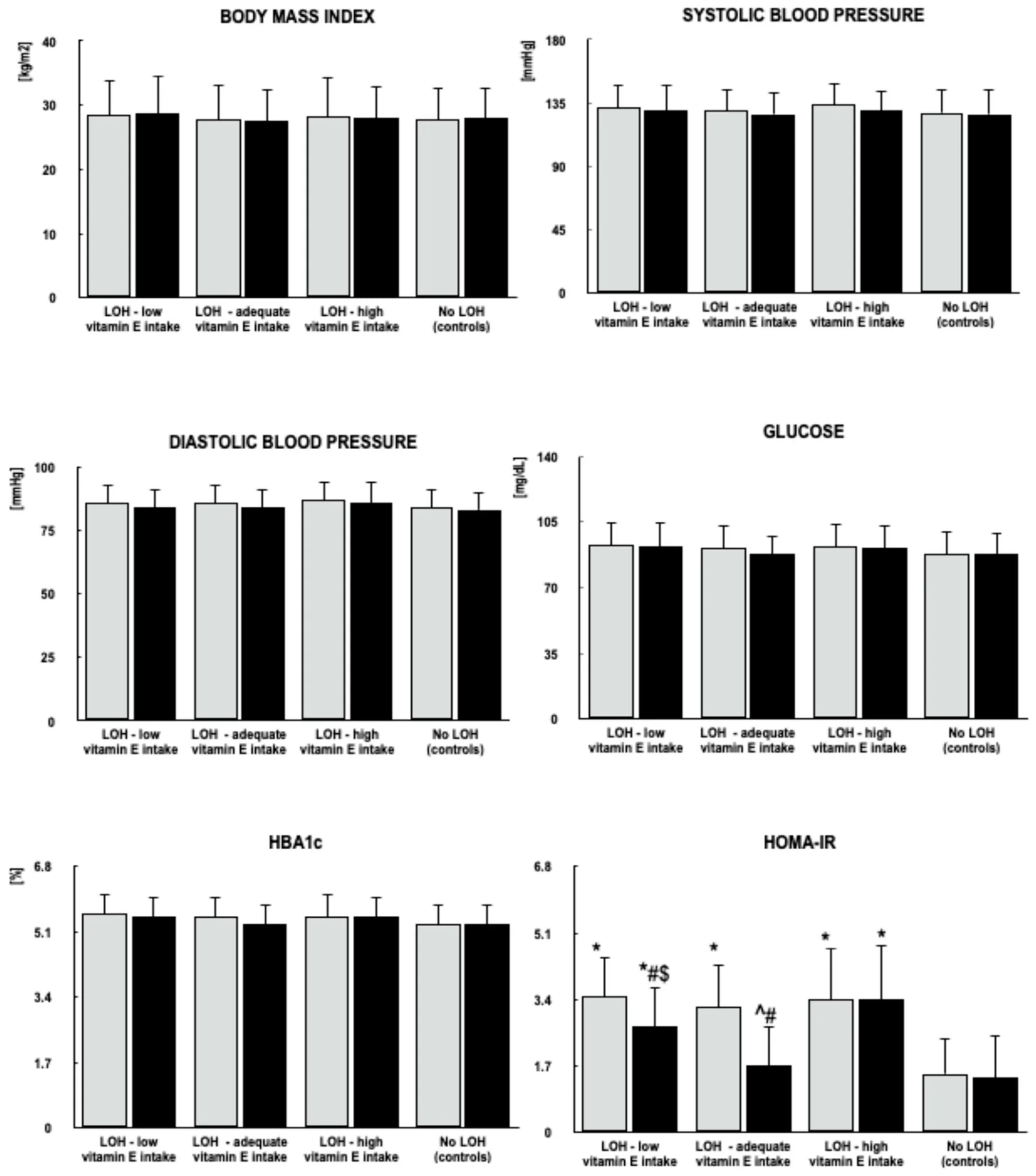
Effects of testosterone replacement on body mass index, blood pressure, and markers of glucose homeostasis in men with LOH, stratified by vitamin E intake. Data are presented as mean and standard deviation. Gray bars represent values at the beginning of the study, while black bars represent values at study completion. Post hoc comparisons: * *p* < 0.05 vs. value at the corresponding time point in men without LOH; ^^^ *p* < 0.05 vs. values at the corresponding time point in men with LOH with low or high vitamin E intake; ^$^ *p* < 0.05 vs. value at the corresponding time point in men with LOH and high vitamin E intake; ^#^ *p* < 0.05 vs. baseline value within the same group. *p*-values were adjusted for multiple comparisons.

**Figure 3:**
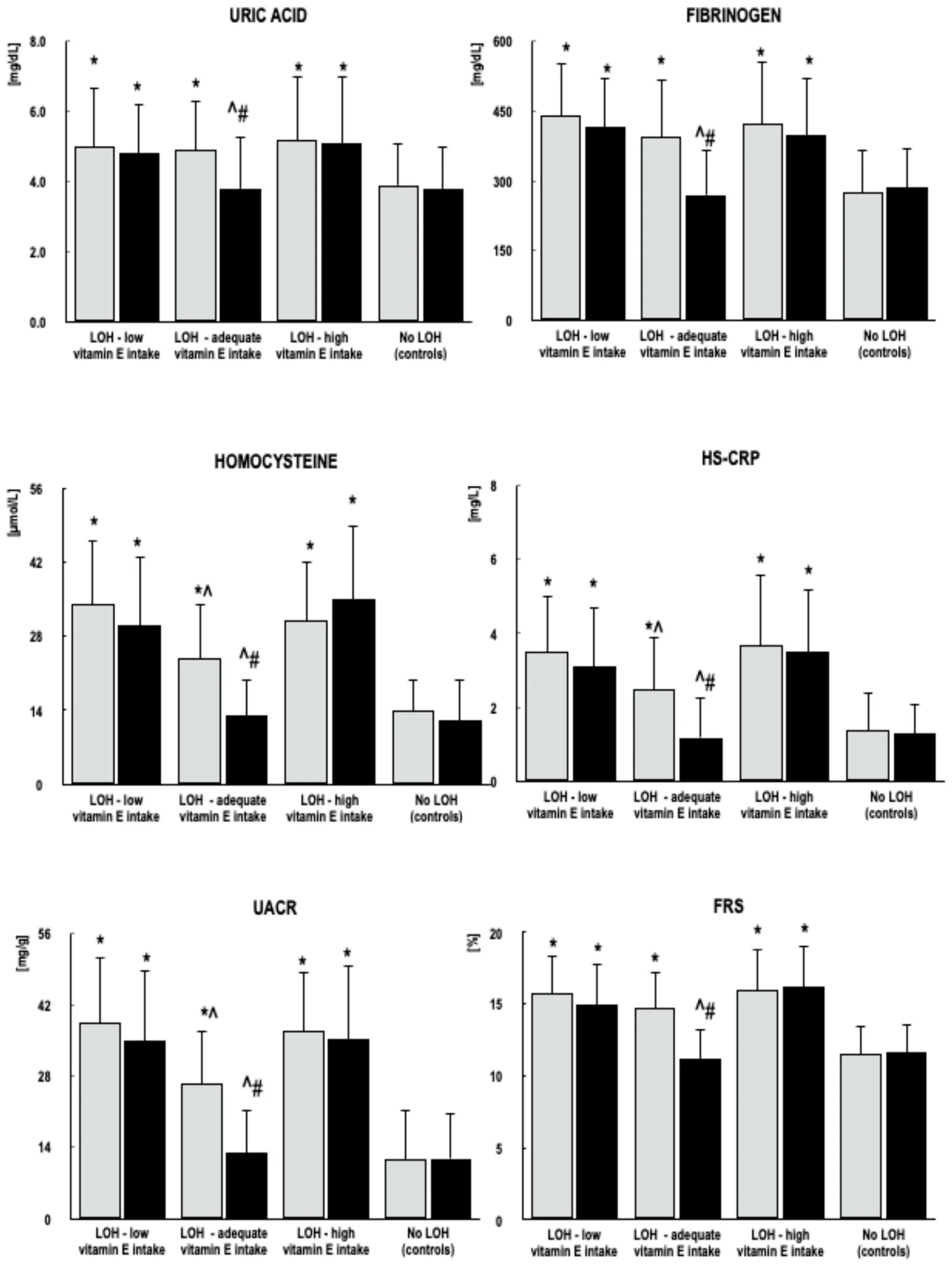
Effects of testosterone replacement on other risk factors and Framingham Risk Score in men with LOH, stratified by vitamin E intake. Data are presented as mean and standard deviation. Gray bars represent values at the beginning of the study, while black bars represent values at study completion. Post hoc comparisons: * *p* < 0.05 vs. value at the corresponding time point in men without LOH; ^^^ *p* < 0.05 vs. values at the corresponding time point in men with LOH with low or high vitamin E intake; ^#^ *p* < 0.05 vs. baseline value within the same group. *p*-values were adjusted for multiple comparisons.

At baseline, with the exception of total daily vitamin E intake and the percentage of vitamin E supplement users, no differences were observed among the study groups with respect to age, smoking status, body mass index, systolic and diastolic blood pressure, plasma levels of 25-hydroxyvitamin D, plasma levels of LH, follicle-stimulating hormone (FSH), thyroid-stimulating hormone (TSH) and free thyroxine, or daily dietary intake of macro- and micronutrients (Table 1). All groups of men with LOH differed from men without LOH with respect to LDL cholesterol, HOMA-IR, uric acid, fibrinogen, homocysteine, high-sensitivity C-reactive protein (hs-CRP), the urinary albumin-to-creatinine ratio (UACR), common carotid intima–media thickness (CIMT), and the Framingham Risk Score (FRS). However, no differences were observed between these groups in total testosterone, fasting glucose, HbA_1c_, total cholesterol, high-density lipoprotein (HDL) cholesterol, or triglycerides. Men with LOH and adequate vitamin E intake differed from those with low or high vitamin E intake in homocysteine, hs-CRP, and UACR. No differences in these parameters were observed between men with LOH and low vitamin E intake and those with high vitamin E intake (Figure 1, Figure 2 and Figure 3).

**Figure 4:**
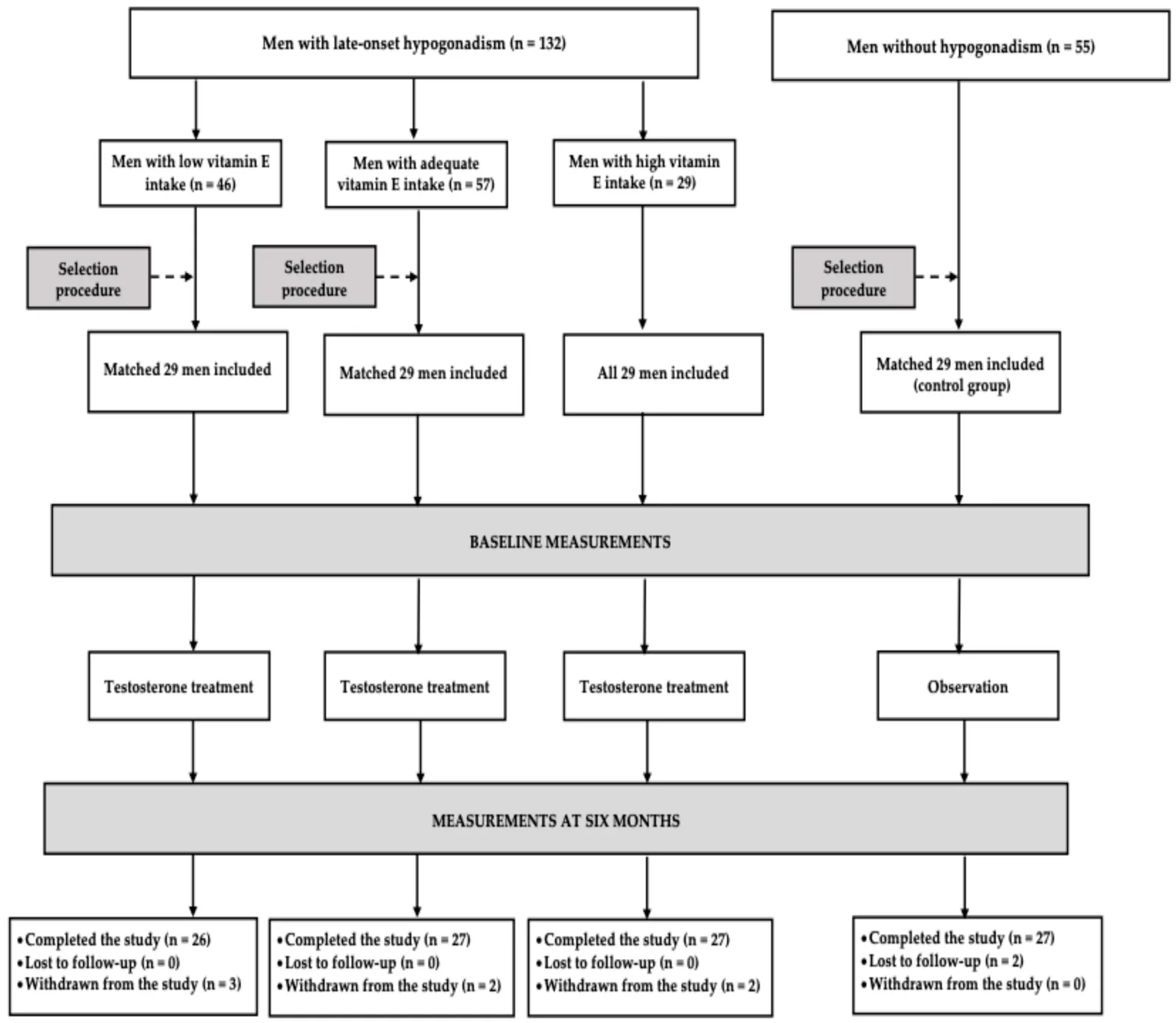
Study participant flowchart.

Of the 87 men with LOH who began participation in the study, 80 completed it, representing 92% of those enrolled. The study was also completed by 27 of the 29 individuals in the control group (93%) (Figure 4). The most common reason for discontinuation was modification of pharmacotherapy, which occurred in four of the testosterone-treated patients (4.6%): two men with LOH and low vitamin E intake (6.9%), one man with LOH and adequate vitamin E intake (3.4%), and one man with LOH and high vitamin E intake (3.4%). Each of the other reasons for withdrawal occurred in one participant (1.1%) and included an increase in hematocrit in a man with LOH and low vitamin E intake; pain and swelling at the injection site requiring a change in the route of testosterone administration in a man with LOH and adequate vitamin E intake; and failure to maintain dietary records in a man with LOH and high vitamin E intake. The two men without LOH who did not complete the study discontinued attendance at follow-up visits without providing a reason (6.9% of the control group) (Supplementary Table S1). Throughout the study period, all patients who completed the study adhered to the dietary recommendations for macronutrient and energy intake and followed the recommended physical activity regimen. Neither the prevalence of smoking nor the mean number of cigarettes smoked changed over the course of the study (Supplementary Figure S1).

The mean vitamin E intake during the study period and at the various assessment time points did not differ significantly from baseline intake or from that observed at other time points within the same group. Throughout the study period, the baseline differences in vitamin E intake between the groups persisted (Table 2). Average intake of other nutrients remained consistent with baseline and was similar across all groups. At study end, 25-hydroxyvitamin D concentrations were 106 ± 19 nmol/L in men with LOH and low vitamin E intake, 102 nmol/L in men with LOH and adequate vitamin E intake, 107 nmol/L in men with LOH and high vitamin E intake, and 111 ± 20 nmol/L in controls, showing no significant change from baseline.

**Figure 5:**
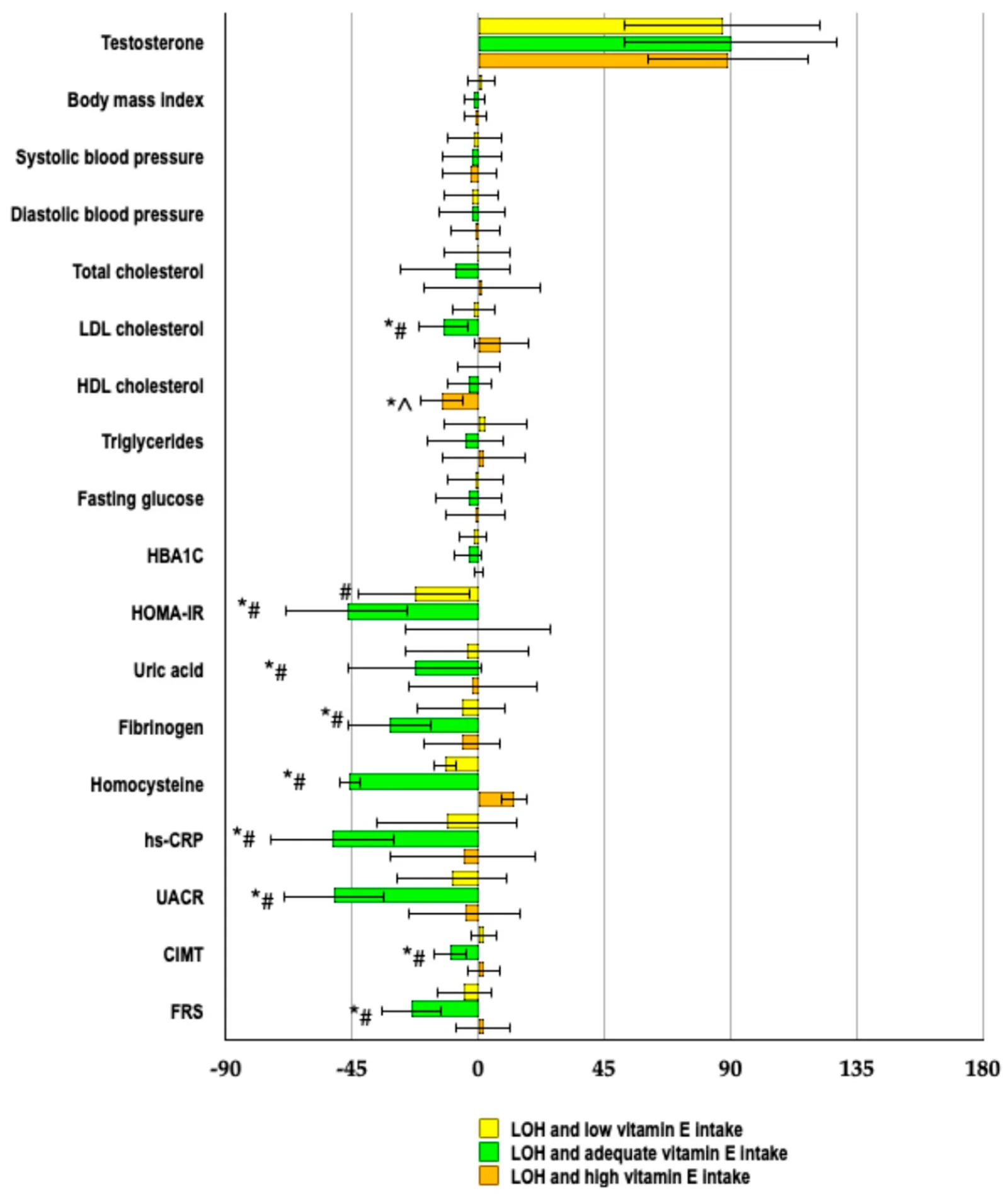
Percentage changes from baseline in studied variables in men with LOH stratified by vitamin E intake. Data are presented as mean ± standard deviation. * *p* < 0.05 vs. LOH and low vitamin E intake; ^^^ *p* < 0.05 vs. LOH and adequate vitamin E intake; ^#^ *p* < 0.05 vs. LOH and high vitamin E intake. *p*-values were adjusted for multiple comparisons.

In each of the testosterone-treated groups, testosterone concentration at the end of the study was higher than at baseline and did not differ from that observed in the control group. No differences were observed among men with LOH and low, adequate, or high vitamin E intake in the magnitude of the increase in testosterone concentration relative to baseline. Testosterone therapy was not associated with changes in total cholesterol or triglyceride concentrations in any analyzed group. Among men with LOH and adequate vitamin E intake, testosterone administration was associated with a reduction in LDL cholesterol levels, an effect not observed in the other testosterone-treated groups. At the end of the study, LDL cholesterol levels in men with LOH and adequate vitamin E intake were lower than those in the other LOH groups. Among men with LOH and high vitamin E intake, a decrease in HDL cholesterol levels was observed, which was not detected in the other groups. At the end of the study, HDL cholesterol levels in these men were lower than those in men without LOH. Although no statistically significant effect of testosterone therapy on CIMT was observed in any group, CIMT at the end of the study was lower in men with LOH and adequate vitamin E intake than in those with low or high vitamin E intake and did not differ from that in men without LOH. Percentage changes from baseline were most pronounced for LDL cholesterol among men with LOH and adequate vitamin E intake, and for HDL cholesterol among men with LOH and high vitamin E intake (Figure 1 and Figure 5; all divergent longitudinal trends across groups were validated by the significant group × time interaction effects provided in Supplementary Table S2).

Testosterone therapy was not associated with changes in body mass index, systolic or diastolic blood pressure, or glucose and HbA_1c_ concentrations. Among men with LOH and low or adequate vitamin E intake, a decrease in HOMA-IR values was observed, which was not noted in men with LOH and high vitamin E intake or in men without LOH. At the end of the study, HOMA-IR values were higher in men with LOH and high vitamin E intake than in men with LOH and low or adequate vitamin E intake and men without LOH. In addition, HOMA-IR values were higher in men with LOH and low vitamin E intake than in those with LOH and adequate vitamin E intake and men without LOH, whereas no difference was observed between men with LOH and adequate vitamin E intake and men without LOH. Percentage changes in HOMA-IR relative to baseline were more pronounced among men with LOH and adequate vitamin E intake than among the other groups of men with LOH (Figure 2 and Figure 5; these distinct longitudinal patterns across the cohorts were statistically confirmed by the group × time interaction analyses detailed in Supplementary Table S2).

Among men with LOH and adequate vitamin E intake, testosterone therapy was associated with reductions in plasma uric acid, fibrinogen, homocysteine, and hs-CRP concentrations, as well as decreased UACR and FRS values. No changes in uric acid, fibrinogen, homocysteine, hs-CRP, UACR, or FRS were observed among men with LOH and low or high vitamin E intake or among men without LOH. At the end of the study, none of these parameters differed between men with LOH and adequate vitamin E intake and men without LOH. In contrast, values in men with LOH and low or high vitamin E intake were higher than those observed in men with LOH and adequate vitamin E intake and men without LOH. Percentage changes in uric acid, fibrinogen, homocysteine, hs-CRP, UACR, and FRS were more pronounced among men with LOH and adequate vitamin E intake than among men with LOH and low or high vitamin E intake (Figure 3 and Figure 5; the observed disparities in chronological trajectories among the study groups were verified by the significant group × time interaction effects documented in Supplementary Table S2).

The proportions of men with retinal arterial sclerotic changes of grades 0–4 at baseline were 31%, 58%, 7%, 4%, and 0% among men with LOH and low vitamin E intake; 37%, 52%, 11%, 0%, and 0% among men with LOH and adequate vitamin E intake; 30%, 56%, 7%, 7%, and 0% among men with LOH and high vitamin E intake; and 63%, 37%, 0%, 0%, and 0% among men without LOH (controls), respectively. At study completion, the corresponding proportions were 23%, 69%, 8%, 0%, and 0% among men with LOH and low vitamin E intake; 52%, 48%, 0%, 0%, and 0% among men with LOH and adequate vitamin E intake; 26%, 59%, 11%, 4%, and 0% among men with LOH and high vitamin E intake; and 59%, 41%, 0%, 0%, and 0% among men without LOH (controls), respectively. Post hoc analysis demonstrated significant baseline differences between men without LOH and each of the three LOH groups (*p* values ranging from 0.006 to 0.035). At study completion, significant differences were observed between men without LOH and men with LOH and low vitamin E intake (*p* = 0.015), as well as between men without LOH and men with LOH and high vitamin E intake (*p* = 0.011). No significant changes in the severity of retinal arterial sclerotic changes were observed within any study group from baseline to study completion (*p* values ranging from 0.085 to 0.906).

At study entry, plasma testosterone levels were inversely correlated with HOMA-IR, LDL cholesterol, fibrinogen, homocysteine, hs-CRP, UACR, and CIMT among men with LOH and low, adequate, or high vitamin E intake (r values ranged from −0.56 to −0.28; adjusted *p*-values ranged from <0.001 to <0.05). Additionally, these cardiometabolic risk markers were correlated with average vitamin E intake among men with LOH and low or high vitamin E intake, with inverse correlations observed in men with low vitamin E intake and positive correlations in those with high vitamin E intake (r values ranged from −0.27 to −0.41 [adjusted *p*-values ranged from <0.01 to <0.05] and from 0.28 to 0.43 [adjusted *p*-values ranged from <0.01 to <0.05], respectively). Changes in LDL cholesterol, HOMA-IR, fibrinogen, homocysteine, hs-CRP, UACR, and CIMT following testosterone therapy were positively associated with changes in plasma testosterone concentrations (Table 3). Among men with LOH and low or high vitamin E intake, changes in these markers were correlated with average vitamin E intake, with positive correlations observed among men with low vitamin E intake and inverse correlations among those with high vitamin E intake (Table 4). Furthermore, among men with LOH and adequate vitamin E intake, changes in FRS following testosterone therapy were positively correlated with changes in homocysteine (r = 0.38, adjusted *p* < 0.01), hs-CRP (r = 0.42, adjusted *p* < 0.01) and UACR (r = 0.40, adjusted *p* < 0.01).

## 3. Discussion

The study included a relatively homogeneous group of patients with LOH, defined as persistent, symptomatic testosterone deficiency in middle-aged or older men in the absence of evidence of a specific organic testicular or hypothalamic–pituitary cause [38]. Given the exclusion criteria applied to eliminate other forms of reproductive axis dysfunction, the findings obtained should therefore be interpreted as pertaining to this specific form of functional male hypogonadism. Regardless of vitamin E intake, all groups of men with LOH exhibited an unfavorable metabolic profile, including reduced insulin sensitivity, a poorer lipid profile, and alterations in other assessed cardiovascular risk factors. This was reflected in a higher estimated cardiovascular risk, as calculated using the FRS. No other measured factors were identified that could explain the observed differences compared with age-matched healthy controls. The study protocol enabled recruitment of a relatively homogeneous patient cohort and minimized the influence of comorbidities and concomitant medications. Moreover, the levels of assessed cardiovascular risk factors were inversely correlated with baseline testosterone concentrations. These findings underscore the importance of early recognition of LOH in middle-aged and older men and highlight the need for further investigation of its metabolic and cardiovascular implications.

An even more noteworthy observation emerging from the present study was that, within the group of patients with LOH, cardiometabolic risk profiles differed according to vitamin E intake. This is supported by baseline differences in homocysteine and hs-CRP concentrations and UACR between individuals with adequate intake and those with either low or high intake. In the case of intake below the EAR, this finding may be consistent with reduced availability of vitamin E and attenuation of its potentially beneficial physiological effects, including its reported antioxidant and anti-atherogenic properties [39,40]. However, what appears even more noteworthy is that a less favorable cardiometabolic risk profile was also observed under conditions of high vitamin E intake. Considering the normal 25-hydroxyvitamin D concentrations and the absence of concomitant differences in the intake of other macro- and micronutrients, several observations may be drawn from these findings. First, the differences between the groups concerned exclusively non-classical cardiovascular risk factors. Therefore, in patients with inadequate vitamin E intake, limiting the assessment solely to classical risk factors—which commonly occurs in clinical practice—may underestimate the actual risk. This finding indicates the potential value of more frequent assessment of non-classical risk factors. Second, both insufficient and high vitamin E intake were associated with a less favorable cardiometabolic profile. Consequently, the findings raise concerns about the uncontrolled use of commercially available preparations containing high doses of vitamin E. Finally, the observed association between vitamin E intake and cardiometabolic risk warrants further investigation, particularly in men with LOH who do not receive hormonal therapy or for whom such therapy is contraindicated.

Our research demonstrated significant differences between the studied cohorts in cardiometabolic outcomes during testosterone replacement, with an inverted U-shaped relationship identified across vitamin E intake levels. The magnitude of the observed changes was greater in the group with adequate intake than in those with low or high intake. With recommended vitamin E intake, all biochemical risk markers that differed significantly from those in the control group at baseline showed changes during testosterone replacement. After six months of treatment, these values were no longer significantly different from those observed in men without LOH. Finally, changes in cardiometabolic risk factors were correlated with the magnitude of the increase in plasma testosterone levels. These findings cannot be attributed to the use of other medications affecting the cardiovascular system, glucose homeostasis, or lipid metabolism, nor to drugs influencing testosterone pharmacokinetics, as such therapies were not permitted. Therefore, these findings describe cardiometabolic changes associated with testosterone treatment in men with LOH, although the observational design does not allow us to determine whether exogenous testosterone caused these changes or whether the response depended on vitamin E intake. Furthermore, the observed differences according to vitamin E intake suggest that optimization of vitamin E intake may enhance the cardiometabolic response to testosterone, although this requires confirmation in further studies.

Vitamin E was the only dietary component whose intake differed between groups. The cohorts were otherwise comparable with respect to plasma 25-hydroxyvitamin D concentrations, which remained unchanged during testosterone treatment, as well as the intake of all other macro- and micronutrients. Similarly, the absence of between-group differences in smoking status, the comparable smoking intensity throughout the study, and statistical adjustment for smoking as a covariate argue against smoking having influenced the observed findings. Further support for an association between the observed changes and vitamin E intake was provided by the presence of correlations between changes in the assessed risk factors and mean vitamin E intake in two of the three cohorts. Nevertheless, the study protocol does not allow for the definitive exclusion of the possibility that the observed differences among the groups could result from imbalances in unmeasured confounding factors, unassessed testosterone-related adverse effects, the presence of asymptomatic or mildly symptomatic comorbidities, or the lack of analysis of data from withdrawn patients at study completion. However, the extent to which these factors influenced the study outcomes cannot be determined from the available data.

A noteworthy finding of this study was the nature of the correlation between changes in the assessed risk factors during testosterone treatment and average vitamin E intake. These correlations were positive in the group with insufficient vitamin E intake, negative in the group with high intake, and not statistically significant in the group with adequate intake. These results suggest that at extremely low and extremely high vitamin E intake levels, cardiometabolic outcomes were most adversely affected, and raise the possibility that even partial modification of vitamin E intake may be associated with more favorable cardiometabolic changes during testosterone treatment, even when intake remains suboptimal. The absence of correlations between dietary content and changes observed during testosterone treatment in the group with adequate intake does not allow us to determine whether there is a precisely defined intake at which testosterone-related cardiometabolic outcomes are optimal. Instead, the findings suggest that cardiometabolic outcomes observed during testosterone treatment may be more favorable across a broad range of vitamin E intake, from 22.4 to 200 IU, achievable through consumption of vitamin E–rich foods or supplementation with low to moderate doses. These findings also raise the possibility that vitamin E intake exceeding the Recommended Daily Allowance (RDA) may be associated with additional cardiometabolic benefits.

High vitamin E intake for health promotion and chronic disease prevention remains a relevant issue in developed countries. Data from the Physicians’ Health Study, conducted among male physicians, demonstrated that 64% of vitamin E supplement users consumed doses of 400 IU or higher [41]. The use of high-dose supplementation was particularly common among age groups comparable to those included in the present study [36]. These data indicate that a substantial proportion of men receiving testosterone therapy may simultaneously consume high doses of vitamin E through supplements available over the counter. In our study, the changes observed during testosterone administration in this group were limited to a potentially unfavorable decrease in HDL cholesterol [42]. This contrasts not only with more pronounced changes in individuals with adequate vitamin E intake but also with the limited, yet still present, metabolic effects observed in individuals with low intake.

Another interesting observation was that, following six months of testosterone treatment, CIMT—considered a marker of early atherosclerotic changes and an independent risk factor for vascular events and cardiovascular mortality [43,44]—no longer differed between men with adequate vitamin E intake and control participants. This finding contrasts with the differences observed at baseline and those that persisted at the end of the study in the other groups of men with LOH. Moreover, at study completion, CIMT also differed between men with adequate vitamin E intake and those with LOH who had either low or high vitamin E intake. In addition, the study groups differed in the severity of retinal vascular sclerotic changes. By the end of the study, the severity of these changes in the group with adequate vitamin E intake no longer differed from that observed in the control group, despite the continued presence of such differences among men with low and high vitamin E intake. These findings are consistent with an association between vitamin E intake and vascular measures observed during testosterone treatment, but they do not establish that vitamin E determines or mediates the anti-atherosclerotic effects of testosterone. No statistically significant changes from baseline were observed during the six-month study, which may reflect the relatively short duration of treatment and the limited reversibility of atherosclerotic vascular changes.

The studied populations of men with LOH had an intermediate risk of both fatal and non-fatal cardiovascular events. Importantly, the results showed lower estimated cardiovascular risk after testosterone treatment in the group with adequate vitamin E intake. Notably, this difference in estimated risk was observed despite a limited impact on the lipid profile, no changes in blood pressure, and no changes in smoking habits—all factors considered in the FRS [45]. Moreover, the observed reduction in estimated cardiovascular risk correlated with decreases in homocysteine, hs-CRP, and UACR, even though these markers are not included in FRS calculations, further supporting their potential predictive value for cardiovascular risk [46,47,48].

The study protocol does not allow for the determination of the mechanisms underlying the observed relationship between vitamin E intake and cardiometabolic outcomes during testosterone treatment. The absence of differences between the studied groups of men with LOH with respect to increases in testosterone concentrations is consistent with the absence of an evident difference in testosterone exposure across the vitamin E intake groups, although it does not exclude pharmacokinetic differences that were not captured by the measurements performed. Interpretation of the results is further complicated by the absence of a specific receptor for this vitamin and by the existence of several distinct forms of vitamin E. Consequently, the findings may be interpreted in several ways. First, the observed association between vitamin E intake and cardiometabolic outcomes may be related to its antioxidant properties. Adequate vitamin E intake could potentially contribute to an enhancement of the modest antioxidant effects of testosterone observed in the setting of impaired testicular function [49]. This explanation is supported by evidence demonstrating a significant role of oxidative stress in determining the lipid profile, insulin sensitivity, low-grade systemic inflammation, increased coagulation, homocysteinuria, and increased urinary albumin excretion [50,51,52]. Second, high amounts of vitamin E may downregulate the androgen receptor, an effect that has so far been demonstrated in prostate cancer cells [53]. Third, an indirect effect of vitamin E on the pregnane X receptor or on receptor activator of nuclear factor κB ligand signaling pathways is also possible [54,55]. Redox-related mechanisms may also provide a potential explanation for the observed differences in cardiometabolic changes during testosterone treatment in men with elevated vitamin E intake. Increased vitamin E availability may promote a shift from antioxidant to pro-oxidant activity through the accumulation of tocopheroxyl radicals, which, in the presence of metabolic disturbances, transition metal exposure, or genetic susceptibility, may enhance oxidative processes [56] and may potentially attenuate the cardiometabolic changes observed during testosterone treatment.

Two measures were taken to minimize potential variability in vitamin E exposure. First, individuals with borderline α-tocopherol intake, as well as those with comorbidities that could affect vitamin E pharmacokinetics or receiving medications that could have such effects, were excluded from the study. Second, dietary vitamin E intake was assessed repeatedly using 7-day food diaries completed before each of the four scheduled follow-up visits, providing four separate weeks of dietary assessment over the six-month study period. Despite significant differences in vitamin E intake between the groups, intake remained relatively stable within each group throughout the study period. Nevertheless, given interindividual differences in intestinal absorption, lipoprotein transport, tissue distribution, storage, and metabolism [57,58], the observed differences in the cardiometabolic effects of testosterone may not necessarily be attributable to differences in reported vitamin E intake. The lack of measurement of circulating α-tocopherol concentrations represents an important limitation of the present study, as lipid-adjusted plasma α-tocopherol concentrations would provide a more informative measure of biological vitamin E availability than dietary vitamin E intake. Therefore, the present findings should be interpreted as an association between vitamin E intake and testosterone responsiveness rather than as evidence of a causal effect of vitamin E on testosterone response. Given the tissue-specific distribution of vitamin E, future studies could also consider directly measuring α-tocopherol content in tissues [57,59].

Several additional methodological limitations of the present study warrant consideration. Although the study exceeded the calculated sample size, the relatively small number of participants limited the strength of the conclusions. Given the cohort design, the findings may have been influenced by selection bias and residual confounding. Residual heterogeneity within the LOH cohorts cannot be excluded, including the possible presence of other forms of hypogonadism. Owing to the broad age distribution, differences between middle-aged and older participants cannot be excluded. Because the study assessed only surrogate endpoints, the results provide probabilistic rather than definitive evidence of clinically meaningful outcomes. The possibility of delayed effects from non-pharmacological interventions cannot be entirely ruled out. Relatively long food-recording periods might have contributed to under-reporting or incomplete data collection. While the protocol sought to control sources of variability in hormone measurements—including diurnal timing, seasonal fluctuations, and analytical factors—the potential contribution of regression toward the mean to the observed findings cannot be completely dismissed [60]. Finally, the study does not provide insight into the molecular mechanisms underlying the observed relationship between vitamin E intake and cardiometabolic outcomes during testosterone treatment.

## 4. Materials and Methods

The study was conducted as a prospective cohort investigation and was approved by the relevant institutional review board. All procedures conformed to the 1964 Declaration of Helsinki and its later amendments. Patient recruitment began in February 2023 and continued for the following 24 months.

### 4.1. Patients

Participants were men aged 40–75 years diagnosed with LOH. Inclusion criteria required total testosterone concentrations below 10.4 nmol/L (3.0 ng/mL) on at least two occasions, separated by no fewer than six weeks, with measurements performed under standardized morning conditions, together with classic symptoms of hypogonadism, including reduced frequency of morning erections, decreased sexual thoughts, and erectile dysfunction. Plasma LH and FSH concentrations were assessed to distinguish primary from secondary hypogonadism. Men with biochemical evidence of primary hypogonadism or a known or suspected organic testicular disorder were excluded. A LH concentration ≥ 9.4 IU/L was considered indicative of primary hypogonadism, in accordance with the European Male Ageing Study classification and subsequent clinical practice guidelines [61,62]. FSH was considered an additional marker of testicular dysfunction and was interpreted together with LH, clinical findings, and medical history; no predefined FSH cutoff was used. Men with low or inappropriately normal LH concentrations were eligible provided that no organic hypothalamic–pituitary disorder was identified during the pre-enrollment evaluation. Serum prolactin was assessed in all patients before enrollment to rule out hyperprolactinemia as a cause of low testosterone levels. Assessment of iron status and other pituitary hormones was performed before enrollment when clinically indicated. Pituitary magnetic resonance imaging was considered in cases of severe testosterone deficiency (<6 nmol/L), additional pituitary hormone abnormalities, or clinical findings suggestive of a sellar lesion. Additional inclusion criteria included adherence to dietary and aerobic physical activity recommendations (consistent with current guidelines [63]) for at least 12 weeks before study enrollment, as well as plasma 25-hydroxyvitamin D concentrations within the predefined range of 75–150 nmol/L. Smoking status was not an exclusion criterion. All smokers were informed about the adverse health effects of tobacco use and advised to quit smoking in accordance with the requirements of the institutional ethics committee and standard medical practice. Only smokers who were unwilling to attempt smoking cessation were eligible for participation, as a change in smoking status could affect the study outcomes. The use of vitamin E supplements was permitted, provided that vitamin E was the sole active ingredient in the preparation.

Based on their mean total vitamin E intake during the two-week period immediately preceding study initiation, patients were assigned to one of three groups according to their vitamin E intake: low, adequate, or high. Dietary vitamin E intake was calculated using food composition tables and expressed as α-tocopherol equivalents (mg α-TE/day) [64]. In patients who did not use vitamin E supplements, total vitamin E intake corresponded to dietary intake from food. In patients who used vitamin E supplements, total vitamin E intake was calculated as the sum of dietary and supplemental intake. The mean intake was calculated as the arithmetic mean of the daily total vitamin E intake recorded for all days during this period. Intake was classified as low if it was below the EAR (12 mg/day) [65]. Adequate intake was defined as mean total vitamin E intake equal to or greater than the RDA (15 mg/day) but not exceeding 200 IU/day [65]. High intake was defined as total intake exceeding 400 IU/day, a threshold associated with increased all-cause mortality in a meta-analysis by Miller et al. [26]. Participants with average vitamin E intakes between 12 and 15 mg/day or between 200 and 400 IU/day were excluded because these ranges represented borderline values for which unequivocal assignment to a predefined intake category could not be ensured. Excluding these participants minimized the potential impact of inadvertent errors in intake estimation or calculation and reduced the risk of misclassification in situations where daily vitamin E intake could fall within different intake categories on different days. This approach was adopted to ensure a more robust and consistent classification of participants into the predefined intake groups. As the relevant intake thresholds were expressed in different units, conversion in both directions was required to apply the predefined intake criteria. Supplemental vitamin E intake was converted from IU to mg α-TE according to the stereochemical form of the supplement, using a conversion factor of 0.67 mg α-TE per IU for RRR-α-tocopherol and its esters (including RRR-α-tocopheryl acetate and succinate), and 0.45 mg α-TE per IU for all-rac-α-tocopherol and its corresponding esters (including all-rac-α-tocopheryl acetate and succinate) [65]. Conversely, when conversion from mg α-TE to IU was required to apply the criteria based on Miller et al. [26], 1 mg α-TE was considered equivalent to 1.49 IU for RRR-α-tocopherol forms and 2.22 IU for all-rac-α-tocopherol forms. The lower biological activity of all-rac-α-tocopherol forms, which contain a mixture of stereoisomers with different biological activities, was accounted for by applying the conversion factor specific to synthetic vitamin E. The control group comprised male relatives of study participants who did not have LOH, met no exclusion criteria, and did not require testosterone treatment.

An a priori sample size analysis was performed based on the anticipated effects of the intervention on the prespecified, co-primary metabolic and cardiovascular outcomes. As no single outcome was considered hierarchically superior to the others, sample size calculations were performed separately for each co-primary outcome, based on the anticipated between-group differences in longitudinal changes from baseline, the expected variability, a statistical power of 80%, and a two-sided significance level of 5%. The final sample size was determined by the outcome requiring the largest number of participants. Among the co-primary outcomes, hsCRP required the largest sample size. A minimum of 24 participants per group was required to detect a 25% between-group difference in the longitudinal change in hsCRP, with 80% statistical power and a two-sided significance level of 5% [15]. To account for potential dropouts, 29 participants were included in each group. Due to the small number of men with high vitamin E intake, all eligible participants with an intake exceeding 400 IU were included in the high vitamin E intake group, whereas participants in the remaining groups were selected from larger pools of eligible candidates to facilitate matching. This selection procedure was designed to ensure that the three groups were comparable with respect to age, smoking status, body mass index, and testosterone levels. To minimize seasonal effects and confounding factors, enrollment occurred year-round, with each group’s seasonal representation ranging from 20% to 30%.

Exclusion criteria included a history of prostate or breast cancer; severe lower urinary tract symptoms (International Prostate Symptom Score > 19); baseline prostate-specific antigen > 4 ng/mL (or >3 ng/mL for individuals at elevated risk of prostate cancer); recent cardiovascular events—including myocardial infarction, acute coronary syndrome, unstable angina, stroke, or coronary revascularization—within six months prior to enrollment; severe heart failure (New York Heart Association class II–IV); uncontrolled hypertension; hematocrit > 50%; or untreated obstructive sleep apnea. Additional exclusions included diabetes mellitus; any disorders of the hypothalamus, pituitary gland, adrenal glands, thyroid gland, or parathyroid glands; renal or hepatic insufficiency; malabsorption syndromes; macro- or micronutrient deficiencies; other chronic medical conditions; and the use of medications affecting cardiovascular risk, reproductive axis activity, or interfering with the pharmacokinetics of testosterone or vitamin E.

### 4.2. Study Design

The study lasted six months, during which participants received intramuscular testosterone injections while maintaining their usual lifestyle. This duration was chosen to limit the burden of participation and the risk of participant dropout and was supported by our previous study, in which it was sufficient to demonstrate differential effects of testosterone therapy on similar endpoints according to vitamin D status [15]. Participants were administered a 250 mg dose of a blended testosterone ester preparation containing 30 mg of testosterone propionate, 60 mg of testosterone phenylpropionate, 60 mg of testosterone isocaproate, and 100 mg of testosterone caproate. Injections were administered every 21 days, with a permissible deviation of up to three days from the scheduled date. Testosterone injections were administered by qualified medical personnel and verified by investigators through documentation in the medical record. Participants using vitamin E supplements were required to maintain an unchanged supplementation regimen throughout the study period, identical to the regimen used before study enrollment.

During the study, vitamin E intake was periodically reassessed at 6, 12, 18, and 24 weeks of testosterone treatment, corresponding to the scheduled follow-up visits. At each assessment, participants recorded all foods consumed during the seven days preceding the respective study visit using individual food diaries. All completed dietary records were reviewed by trained investigators for completeness, internal consistency, and missing or ambiguous entries. When necessary, participants were asked to clarify discrepancies or provide additional information to minimize recording errors and ensure the quality of the dietary data. The intake of individual dietary components at each follow-up assessment was calculated using established food composition references based on the type, composition, and quantity of foods consumed [64]. At baseline, vitamin E intake included both dietary intake and, when applicable, intake from supplements. At each follow-up visit, daily intake of vitamin E and other dietary components was determined as the arithmetic mean of the values recorded over the preceding seven-day period. Mean intake during testosterone treatment was subsequently calculated from the values obtained at the four follow-up visits. The baseline assessment was not included in these calculations, as it was used solely for patient categorization.

Follow-up visits were scheduled at six-week intervals and served to evaluate treatment safety, patient adherence to the prescribed therapy, and adherence to recommended nonpharmacological interventions. Even a single missed testosterone dose or administration outside the scheduled dosing window constituted grounds for exclusion. Changes in the dosing of any concomitant medications were not permitted. Short-term (≤10 days) use of antimicrobials, nonsteroidal anti-inflammatory drugs, acetaminophen, antitussives, antidiarrheal agents, laxatives, or soporifics was allowed only if treatment had been discontinued at least four weeks prior to study completion. At each follow-up visit, in accordance with the requirements of the institutional ethics committee, smokers were reminded of the adverse health effects of tobacco use and advised to quit smoking. Participants were informed that, should they quit smoking during the study, they would no longer meet the predefined study criteria and would therefore be withdrawn from the study, without any impact on their medical care.

Participants were withdrawn from the study in the event of serious adverse events, as defined by the Food and Drug Administration, including death, a life-threatening event, hospitalization or prolongation of hospitalization, persistent or significant disability or incapacity, or other medically important events requiring medical or surgical intervention [66]; hematocrit > 54%; an increase in prostate-specific antigen concentration of >1.4 ng/mL from baseline; severe local injection-site reactions requiring a change in the route of testosterone administration; any changes in pharmacological treatment not permitted under the study protocol; failure to adhere to the study protocol; or withdrawal of informed consent.

### 4.3. Measurements

Body mass index was calculated by dividing body weight (kg) by height (m) squared. Blood pressure was measured using a cuff placed around the upper arm at heart level while the patient was seated with the arm supported. The first Korotkoff sound (phase I) was considered the systolic pressure, and the disappearance of the sounds (phase V) the diastolic pressure. CIMT was assessed in the longitudinal plane by the same investigator (one of the coauthors), approximately 10 mm proximal to the carotid bifurcation, defined as the distance from the intima–lumen interface to the media–adventitia boundary. Measurements were obtained bilaterally using high-resolution ultrasonography (Toshiba Aplio-500, Otarawa, Japan), and the mean CIMT was calculated as the average of the left and right sides. Fundus examinations were performed by ophthalmologists collaborating with our research team at baseline and at the end of the study. Retinal arteriolar changes were assessed using the Scheie classification, an ordinal, semi-quantitative grading system for evaluating retinal vascular changes associated with arteriosclerosis [67]. The scale ranges from grade 0 to grade 4, with higher grades indicating progressively more severe vascular abnormalities. Grade 0 indicates the absence of detectable changes, grade 1 represents mild arteriolar wall thickening and an increased light reflex, grade 2 reflects more pronounced arteriolar narrowing and arteriovenous crossing abnormalities, grade 3 is characterized by a “copper wiring” appearance, and grade 4 indicates advanced arteriolar sclerosis with a “silver wiring” appearance [67].

### 4.4. Laboratory Assays

All laboratory measurements were performed at baseline (prior to testosterone administration) and after six months of treatment. Blood samples were collected from the antecubital vein in a separate air-conditioned room between 7:30 and 9:00 a.m. after a 12 h overnight fast. To minimize preanalytical variability, all samples were collected under standardized conditions. Laboratory analyses were performed by a technician blinded to participants’ treatment group allocation. For biochemical and hormonal analyses, venous blood samples were collected into lithium heparin tubes and processed immediately after venipuncture. Blood samples for coagulation testing were collected into tubes containing 3.2% sodium citrate (9:1 blood-to-anticoagulant ratio). Heparinized plasma and citrated plasma were obtained by centrifugation at 2000× *g* for 15 min at room temperature. Whole blood samples collected into EDTA-containing tubes were used for HbA_1c_ determination.

All measurements were performed in duplicate, and the mean value of duplicate measurements was used for statistical analyses. Plasma concentrations of glucose, lipids, and uric acid, urinary concentrations of albumin and creatinine, and whole blood HbA_1c_ concentrations were quantified using the COBAS Integra automated analyzer (Roche Diagnostics, Basel, Switzerland). Insulin, total testosterone, gonadotropins, homocysteine, and 25-hydroxyvitamin D concentrations were measured using direct chemiluminescent immunoassays based on acridinium ester technology (ADVIA Centaur XP, Siemens Healthcare Diagnostics, Munich, Germany). Fibrinogen concentration was determined using the Clauss method with an automated coagulation analyzer (Siemens Healthcare, Warsaw, Poland). hs-CRP concentrations were measured using a chemiluminescent immunoassay (Immulite 2000XPi, Siemens Healthcare, Warsaw, Poland). HOMA1-IR was calculated by multiplying fasting plasma glucose (mg/dL) by fasting insulin (mU/L) and dividing the product by 405. UACR was calculated as urinary albumin concentration divided by urinary creatinine concentration and expressed in mg/g. FRS was calculated according to the established algorithm to estimate the 10-year risk of cardiovascular disease [68].

Analytical performance was assessed using commercially available quality-control materials at defined concentration levels. Assay precision was evaluated by calculating intra-assay and inter-assay coefficients of variation (CV, %). Analytical sensitivity, expressed as the limit of detection, and analytical measurement ranges were determined according to manufacturer specifications and laboratory validation procedures. Detailed analytical characteristics of individual assays are provided in Supplementary Table S3.

### 4.5. Statistical Analysis

The assumptions of residual normality and homogeneity of variance were assessed using the Shapiro–Wilk and Levene’s tests, respectively. Continuous variables, including age, body mass index, blood pressure, glucose, HbA_1c_, total cholesterol, LDL cholesterol, and HDL cholesterol, were analyzed on the original measurement scale because logarithmic transformation was not considered necessary. Other continuous variables showing substantial right-skewness or for which logarithmic transformation improved variance homogeneity were log-transformed to improve distributional properties and stabilize variance. Potential outliers were assessed based on biological plausibility and measurement validity. Observations considered biologically plausible and without evidence of measurement error were retained in the analyses. Baseline analyses included all participants enrolled in the study. Analyses involving measurements at study completion, including between-group comparisons of end-of-study values and within-group comparisons of baseline and end-of-study values, were restricted to participants who completed the study protocol and had available data at study completion. The numbers of participants included at each stage are presented in Figure 4. Baseline characteristics were compared among study groups using analysis of covariance (ANCOVA), with smoking status included as a covariate. For outcomes assessed at baseline and at study completion, longitudinal changes over time and differences between groups were evaluated using repeated-measures ANCOVA, with time (baseline and study completion) as the within-subject factor, treatment group as the between-subject factor, and smoking status as a covariate. The group × time interaction was considered the primary parameter for determining whether the magnitude of change over the study period differed among treatment groups. When significant effects were detected, pairwise post hoc comparisons were performed with Bonferroni adjustment. For variables assessed at multiple time points during the study, longitudinal analyses were performed using repeated-measures ANCOVA, with treatment group, time, and the group × time interaction included in the model. Between-group differences in end-of-study values were additionally assessed using ANCOVA models adjusted for the corresponding baseline values and smoking status. Percentage changes from baseline were calculated for each outcome measure and compared among groups using ANCOVA models with appropriate covariate adjustment, followed by Bonferroni-adjusted pairwise comparisons. Baseline categorical characteristics were compared using the chi-square test. When significant differences were detected, post hoc pairwise comparisons were performed. Changes in disease severity within each study group were evaluated using the Wilcoxon signed-rank test for paired observations. The strength and direction of associations between continuous variables were assessed using Pearson correlation coefficients. To account for the increased risk of false-positive findings associated with multiple hypothesis testing, the Benjamini–Hochberg procedure was used to control the false discovery rate, with adjusted *p*-values below 0.05 considered statistically significant.

## 5. Conclusions

Although increases in the concentrations or urinary excretion of the assessed cardiometabolic risk factors were observed in all groups of men with LOH, for most non-traditional factors—homocysteine, hs-CRP, and UACR—these increases were more pronounced in the presence of inadequate (either low or high) vitamin E intake. Differences in vitamin E intake were also associated with differences in cardiometabolic outcomes observed during testosterone therapy. After six months of intramuscular testosterone ester administration, improvements in most assessed risk factors were observed among men with adequate vitamin E intake. In the group with low vitamin E intake, changes were limited to insulin resistance, whereas in men consuming more than 400 IU daily, no significant changes were observed except for a decrease in HDL cholesterol. These results are consistent with previous observations of increased cardiometabolic risk in men with LOH and indicate an association between vitamin E intake and cardiometabolic outcomes observed during testosterone therapy. However, given the pilot nature of this study, the limitations of the applied protocol, and the absence of an untreated LOH group, these findings do not establish a causal effect of vitamin E intake on the response to testosterone therapy. The findings require confirmation in larger, longer-term clinical trials. Furthermore, the mechanisms underlying the observed differences in cardiometabolic outcomes among groups with varying vitamin E intake warrant further investigation.

## Figures and Tables

**Table 1:** Baseline characteristics of study participants (intention-to-treat analysis) ^1^.

Variable	LOH and Low Vitamin E Intake	LOH and Adequate Vitamin E Intake	LOH and High Vitamin E Intake	No LOH (Controls)
Number	29	29	29	29
Age (years)	60 ± 10	59 ± 9	61 ± 10	59 ± 10
Smokers (%)/Number of cigarettes a day (n)/Duration of smoking (years)	48/10 ± 6/32 ± 12	48/9 ± 6/30 ± 11	52/9 ± 5/32 ± 14	45/10 ± 5/31 ± 15
Body mass index (kg/m^2^)	28.6 ± 5.7	27.8 ± 5.5	28.2 ± 5.9	27.9 ± 4.8
Systolic blood pressure (mmHg)	132 ± 16	131 ± 14	134 ± 14	128 ± 16
Diastolic blood pressure (mmHg)	85 ± 7	84 ± 8	86 ± 8	83 ± 8
Daily vitamin D intake (µg)	19 ± 9	20 ± 8	21 ± 9	22 ± 9
25-hydroxyvitamin D (nmol/L)	102 ± 23	104 ± 22	107 ± 21	109 ± 21
LH (IU/L)	3.5 ± 2.6	3.1 ± 2.2	3.4 ± 2.5	4.0 ± 2.0
FSH (IU/L)	3.4 ± 2.4	3.1 ± 2.6	3.2 ± 2.1	3.9 ± 2.2
TSH (mIU/L)	1.2 ± 1.0	1.1 ± 0.9	1.3 ± 1.1	1.2 ± 0.9
Free thyroxine (pmol/L)	15.1 ± 2.9	14.8 ± 2.7	15.3 ± 3.0	15.6 ± 2.5
Total daily vitamin E intake (dietary sources and supplements) (mg α-TE) ^2^	7 ± 3	40 ± 48 *	564 ± 162 *^^^	29 ± 25 *^#^
Vitamin E supplement use (%)	0	48 *	100 *^^^	38 *^#^
Daily selenium intake (µg)	72 ± 15	76 ± 20	78 ± 23	75 ± 18
Daily inositol intake (mg)	695 ± 208	725 ± 320	805 ± 355	760 ± 296
Daily vitamin A intake (µg)	955 ± 400	985 ± 384	1105 ± 510	1023 ± 465
Daily lipid intake (g)	108 ± 28	106 ± 30	110 ± 32	102 ± 29
Daily protein intake (g)	94 ± 20	101 ± 25	91 ± 26	98 ± 21
Daily carbohydrate intake (g)	300 ± 60	288 ± 70	278 ± 80	291 ± 58
Daily alcohol intake (g)	30 ± 16	28 ± 14	26 ± 15	24 ± 18
Daily fiber intake (g)	18 ± 8	20 ± 8	22 ± 10	20 ± 9

Data are presented as mean ± standard deviation, unless otherwise specified. ^1^ This analysis includes the entire cohort of initially enrolled participants, regardless of subsequent dropouts. ^2^ Vitamin E intake expressed in international units—LOH and low vitamin E intake: 10.4 ± 4.4 IU, LOH and adequate vitamin E intake: 65.5 ± 78.6 IU, LOH and high vitamin E intake: 973 ± 280 IU, No LOH: 45.3 ± 39.1 IU. Post hoc comparisons: * *p* < 0.05 vs. LOH and low vitamin E intake; ^^^ *p* < 0.05 vs. LOH and adequate vitamin E intake; ^#^ *p* < 0.05 vs. LOH and high vitamin E intake. *p*-values were adjusted for multiple comparisons.

**Table 2:** Vitamin E intake in study groups during the study period.

Group	Before the Study	During the Study				
		Week 6	Week 12	Week 18	Study End	Mean Intake
LOH and low vitamin E intake	7 ± 3	8 ± 2	7 ± 3	7 ± 2	7 ± 2	8 ± 2
LOH and adequate vitamin E intake	40 ± 48 *	43 ± 37 *	36 ± 40 *	41 ± 35 *	37 ± 42 *	39 ± 40 *
LOH and high vitamin E intake	564 ± 162 *^^^	571 ± 175 *^^^	547 ± 168 *^^^	545 ± 171 *^^^	555 ± 179 *^^^	554 ± 208 *^^^
No LOH (controls)	29 ± 25 *^#^	30 ± 22 *^#^	25 ± 21 *^#^	27 ± 24 *^#^	26 ± 26 *^#^	27 ± 28 mg *^#^

Data are presented as mean ± standard deviation and expressed as mg α-TE/day. The mean vitamin E intake was calculated as the arithmetic mean of values recorded at all assessed time points, excluding the baseline assessment. Post hoc comparisons: * *p* < 0.05 vs. LOH and low vitamin E intake; ^^^ *p* < 0.05 vs. LOH and adequate vitamin E intake; ^#^ *p* < 0.05 vs. LOH and high vitamin E intake. *p*-values were adjusted for multiple comparisons.

**Table 3:** Correlations between the effect of testosterone on cardiometabolic risk factors and changes in plasma testosterone concentrations in patients with LOH.

Variable	LOH and Low Vitamin E Intake	LOH and Adequate Vitamin E Intake	LOH and High Vitamin E Intake
Δ LDL cholesterol	0.40 **	0.49 ***	0.34 *
Δ HOMA-IR	0.37 **	0.51 ***	0.30 *
Δ fibrinogen	0.35 *	0.48 ***	0.32 *
Δ homocysteine	0.38 *	0.58 ***	0.35 *
Δ hs-CRP	0.40 **	0.55 ***	0.34 *
Δ UACR	0.34 *	0.52 ***	0.30 *
Δ CIMT	0.28 *	0.47 **	0.26 *

The data represent correlation coefficients (r values). * *p* < 0.05, ** *p* < 0.01, and *** *p* < 0.001. *p*-values were adjusted for multiple comparisons.

**Table 4:** Correlations between the effect of testosterone on cardiometabolic risk factors and average vitamin E intake in groups of patients with LOH with low or high intake of this vitamin.

Variable	LOH and Low Vitamin E Intake	LOH and High Vitamin E Intake
Δ LDL cholesterol	0.43 **	−0.41 **
Δ HOMA-IR	0.46 **	−0.37 **
Δ fibrinogen	0.44 **	−0.34 *
Δ homocysteine	0.50 ***	−0.49 ***
Δ hs-CRP	0.60 ***	−0.46 **
Δ UACR	0.56 ***	−0.47 ***
Δ CIMT	0.35 *	−0.29 *

The data represent correlation coefficients (r values). * *p* < 0.05, ** *p* < 0.01, and *** *p* < 0.001. *p*-values were adjusted for multiple comparisons.

## Data Availability

The data pertaining to this study is contained within the article. Further inquiries can be directed to the corresponding author.

## Supplementary Materials

The following supporting information can be downloaded at: https://www.mdpi.com/article/10.3390/ijms27208953/s1.
