# Supplementary material for: The Impact of Vitamin E Intake on Cardiometabolic Outcomes of Testosterone Replacement Therapy in Men with Late-Onset Hypogonadism
Source: Int J Mol Sci. 2026 Oct 9;27(20):8953. doi: 10.3390/ijms27208953 (PMC13704612; doi:10.3390/ijms27208953)
Supplement: Supplementary file 1 [file ijms-27-08953-s001.zip › ijms-4587810-supplementary.pdf]

Supplementary Table S1. Individual reasons for premature study discontinuation.

LOH - low vitamin E intake	LOH - adequate vitamin E intake	LOH - high vitamin E intake	No LOH (controls)
New medication initiated (cetirizine) (at Week 6)	New medication initiated (mebeverine) (at Week 12)	New medication initiated (paroxetine) (at Week 12)	Lost to follow-up; no reason provided (at Week 12)
New medication initiated (latanoprost) (at Week 18)	Moderate pain and localized swelling of the left gluteal muscle at the testosterone injection site; testosterone injections replaced with topical testosterone cream (at Week 6)	Diet records not completed (at study end)	Lost to follow-up; no reason provided (at Week 18)
Hematocrit increased to 55% (at Week 12)			

Supplementary Table S2. Statistical evaluation of group × time interaction effects on patient clinical outcomes.

Variable	F(3, 102)	Partial η²	Adjusted p-value
**Testosterone**	**61.06**	**0.64**	**< 0.001**
**Total cholesterol**	**2.56**	**0.07**	**0.060**
**LDL cholesterol**	**6.93**	**0.17**	**< 0.001**
**HDL cholesterol**	**2.79**	**0.08**	**0.042**
**Triglycerides**	**0.24**	**0.01**	**0.871**
**Body mass index**	**0.77**	**0.02**	**0.515**
**Systolic blood pressure**	**0.21**	**0.01**	**0.887**
**Diastolic blood pressure**	**0.20**	**0.01**	**0.895**
**Glucose**	**0.41**	**0.01**	**0.749**
**HBA1c**	**1.16**	**0.03**	**0.328**
**HOMA-IR**	**12.68**	**0.27**	**< 0.001**
**Uric acid**	**3.41**	**0.09**	**0.022**
**Fibrinogen**	**9.18**	**0.21**	**0.001**
**Homocysteine**	**11.34**	**0.25**	**< 0.001**
**hsCRP**	**4.98**	**0.14**	**0.003**
**UACR**	**9.38**	**0.20**	**< 0.001**
**FRS**	**16.02**	**0.32**	**< 0.001**
**CIMT**	**2.72**	**0.07**	**0.048**

Supplementary Table S3. Analytical characteristics of individual assays.

Compound	Limit of detection	Analytical measuring range	Intra-assay precision (CV, %)	Inter-assay precision (CV, %)
**Testosterone**	**2 ng/dL**	**10–1500 ng/dL**	**5.1**	**7.6**
**LH**	**0.1 IU/mL**	**0.3-200 IU/mL**	**3.5**	**4.2**
**FSH**	**0.2 IU/mL**	**0.3-200 IU/mL**	**3.2**	**4.0**
**Total cholesterol**	**1.0 mg/dL**	**10–800 mg/dL**	**1.4**	**2.0**
**LDL cholesterol**	**1.0 mg/dL**	**5–400 mg/dL**	**2.6**	**3.9**
**HDL cholesterol**	**0.5 mg/dL**	**3–120 mg/dL**	**2.0**	**3.1**
**Triglycerides**	**1.0 mg/dL**	**10–1000 mg/dL**	**1.8**	**2.8**
**Glucose**	**0.5 mg/dL**	**2–750 mg/dL**	**1.2**	**1.8**
**HBA1c**	**0.05 %**	**4.0–15.0 %**	**1.5**	**2.3**
**Insulin**	**0.1 mIU/L**	**0.5–300 mIU/L**	**4.1**	**6.1**
**Uric acid**	**0.1 mg/dL**	**0.2–25 mg/dL**	**1.8**	**2.4**
**Fibrinogen**	**10 mg/dL**	**30–700 mg/dL**	**3.2**	**5.2**
**Homocysteine**	**0.5 μmol/L**	**1–65 μmol/L**	**4.1**	**6.1**
**hsCRP**	**0.02 mg/L**	**0.1–150 mg/L**	**5.2**	**7.1**
**25-hydroxyvitamin D**	**2.5 nmol/L**	**10–375 nmol/L**	**7.1**	**9.8**
**Urinary albumins**	**1.0 mg/dL**	**3–400 mg/L**	**3.1**	**4.5**
**Urinary creatinine**	**0.05 mg/dL**	**5–300 mg/dL**	**1.7**	**2.4**

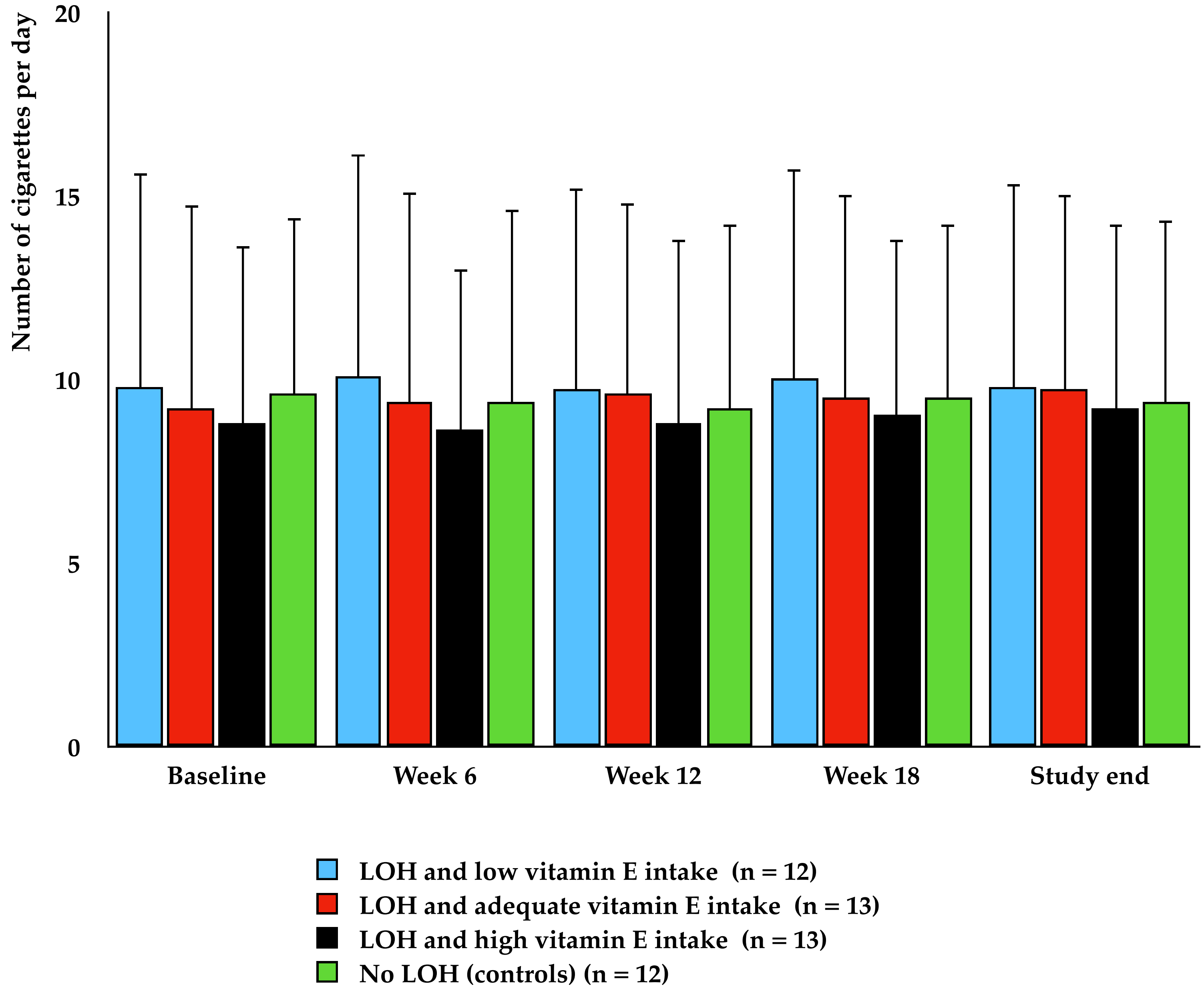

Supplementary Figure S1. Daily number of cigarettes smoked by smokers participating in the study. Data are presented as mean and standard deviation. The number of smokers who completed the study in each group is indicated in parentheses.
